# Effect of Olive Oil Hydrogel as a Fat Replacer in Beef Meatballs

**DOI:** 10.17113/ftb.62.01.24.8134

**Published:** 2024-03

**Authors:** Recep Palamutoğlu, Cemal Kasnak, Buket Özen Ünaldı, Sabire Duman, Ayşegül Türk Baydır

**Affiliations:** 1 Afyonkarahisar Health Sciences University, Department of Nutrition and Dietetics, Dörtyol 2078, 03030 Afyonkarahisar, Turkey; 2 Afyon Kocatepe University, Food Control Research and Application Center, Gazlıgöl, 03200 Afyonkarahisar, Turkey

**Keywords:** fat replacement, gelatine, hydrogel, meatball, olive oil

## Abstract

**Research background:**

Meat and meat products are essential sources of dietary saturated fatty acids. However, excessive consumption of meat and meat products may be harmful to human health. The study evaluates the effect of fat replacement with hydrogels (olive oil in water emulsions gelled by gelatine) in meatballs.

**Experimental approach:**

The effect of replacing fat with different ratios of hydrogel (control, 25 (F25), 50 (F50), 75 (F75) and 100 % (F100)) on the chemical (fatty acids and thiobarbituric acid reactive substances (TBARS)) and physical (cooking loss, diameter reduction, fat retention, water retention, colour and texture analysis) characteristics of the meatballs were analyzed.

**Results and conclusions:**

The fat content of raw meatball samples was reduced from (31.2±2.2) to (10.5±0.4) % in the sample with the highest fat substitution (F100). The energy levels of the F100 samples were almost 56 % lower than of the control group. Monounsaturated fatty acids (MUFAs) represented the dominant group in all substitution rates of the meatballs, followed by saturated fatty acids (SFAs) and finally polyunsaturated fatty acids (PUFAs). Among the raw meatball samples, the highest oxidation occurred in the F50 and F100 groups. However, it was determined that the difference between F25 and F75 and the difference between control and F75 were not statistically significant (p˃0.05). When the cooked samples were compared, the highest thiobarbituric acid (TBA) value was found in the F50 sample, followed by the F100 and F75 samples. The difference between the mean values of springiness and cohesiveness of the samples was not significant (p˃0.05). The hardness value of samples decreased significantly (p˂0.001) with >75 % fat replacement.

**Novelty and scientific contribution:**

It can be concluded that the oil replacement rate that may satisfy consumer demand without impairing the product technological and chemical quality should be <75 %. As the fat replacement ratio increases, the SFA content of cooked meatballs decreases, while the MUFA and PUFA contents increase. Considering the positive effects of reducing the intake of SFAs and increasing the intake of unsaturated fatty acids on non-communicable diseases such as cardiovascular diseases, fat replacement in meatballs is important for future developments.

## INTRODUCTION

Meat and meat products are an important food group in the population’s diet as they contain macro- and micronutrients such as protein, fat, vitamin B12, iron and zinc (*1**,**2*). Meatballs are the most produced meat product in Turkey. It is followed by sucuk (a fermented meat product), kavurma (a cooked meat product), doner kebab (a minced meat product), salami and sausage (*3*). There are approx. 290 types of meatballs produced and consumed in Turkey, which vary according to region and chef. The type of meat and fat used in the production, different ingredients added to the meatball mix (spices, bread, *etc.*), technological processes and meat cooking techniques are the main reasons for these differences (*4*). Excessive consumption of these processed meat products, which contain 20–30 % animal fat (*5*), might be harmful to human health. The high amount of saturated fatty acids in the diet is closely related to low-density lipoprotein cholesterol concentrations, a marker of cardiovascular disease (*6*). Meat products stand out as an important source of dietary saturated fatty acids. For this reason, there is an increasing research interest in reducing the saturated fatty acid content while protecting other sensory properties (*1*). In reformulation studies of meat products, two main approaches considering the lipid content are: (*i*) lowering the amount of lipids, and (*ii*) modifying the lipid composition. Conventional strategies applied to meat products include protein-based replacers, cereal flour, pre-emulsions and dietary fibre. Today, new strategies have been developed, which include oleogels and emulsion gels (*7*). Since the oil affects some technological properties of the product, simply reducing the amount of oil or modifying its composition does not entirely serve this purpose in this type of product (flavour, juiciness, texture, heat transfer, *etc.*) (*5*).

Emulsions with a network structure resembling a gel and textural characteristics resembling solids are referred to as gelled emulsions (*7*). Gelled emulsions, as compared to oil-in-water emulsions, may be a better option for mimicking the hardness and water-holding capacity of lard, which is currently used in most meat products (*8*). Structured fat replacers such as oleogels (*9**,**10*) and hydrogels (*11**-**13*) are being further researched to achieve these goals. Additionally, the use of fat substitutes can lead to engineering problems in some cases because the flavour and texture of food are significantly influenced by the fat in the food composition.

Olive oil contains 55–83 % oleic acid, 3.5–21 % linoleic acid, 7.5–20 % palmitic acid, 0.5–5 % stearic acid and 0.1–1.5 % linolenic acid, as well as significant quantities of bioactive compounds. Its composition is known to have a beneficial effect on the prevention of cardiovascular disease, cancer and diabetes (*14**,**15*).

The study aims to investigate the lipid reformulation of meatballs by replacing 25, 50, 75 and 100 % beef fat with gelled olive oil-water emulsion. In the study, the technological, physicochemical and nutritional properties of raw and cooked meatballs were determined.

## MATERIALS AND METHODS

Butylated hydroxyanisole (BHA), hexane, chloroform, glacial acetic acid, methanol, perchloric acid, tetramethoxypropane and thiobarbituric acid (TBA) reagents were purchased from Sigma-Aldrich, Merck (St. Louis, MO, USA).

### Preparation of hydrogel emulsions

**Fig. S1:**
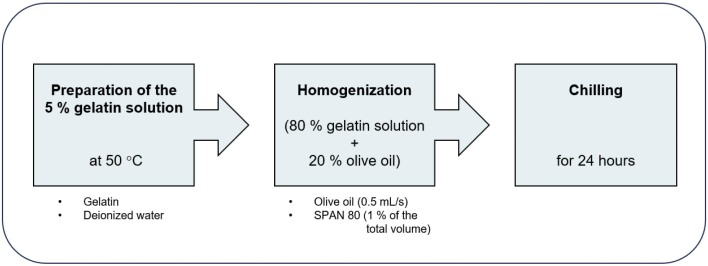
Flow chart for the preparation of hydrogel emulsion

The following formulation was used to produce hydrogel emulsions (Fig. S1): 80 % gelatine (Alfasol, Kimbiotek, Istanbul, Turkey) solution and 20 % olive oil (Savola Gida San As., Balikesir, Turkey). The gelatine used was a 5 % solution. SPAN80 (Sigma-Aldrich, Merck) was added at 1 % of the total volume. At 50 °C, gelatine was dissolved in deionized water to make a transparent solution. The gelatine solution was then homogenized at 16 000 rpm for 3 min with the addition of olive oil at a rate of 0.5 mL/s (Wisd HG-15D homogenizer; Daihan Scientific, Gangwon-do, Republic of Korea). The resulting mixture was then chilled for 24 h.

### Preparation of meatballs

Meat and fat were purchased from a local butcher in Afyonkarahisar, Turkey. The neck muscles and fat (intestinal adipose tissue) were obtained from 2-year-old Simmental cattle that were fed in the Afyonkarahisar province. The muscles and fat were obtained 24 h after the *rigor mortis*. Muscles and fat were comminuted separately at 0 °C using a 6- and 3-mm plate (Mateka EPA 22T; Istanbul, Turkey) and then cold-transported to the laboratory in 10 min. They were kept cold until the meatballs were prepared.

Meat 75 % and fat 25 % were used for the preparation of meatballs. To eliminate the influence of spices on the taste and odour perception, only salt was added (*w*=2 %) (*10*). The animal fat in the mixture was replaced by 25, 50, 75 and 100 % of prepared hydrogel forming five meatball groups: control, F25, F50, F75 and F100. Meatballs were prepared by rolling pieces of 25 g from the prepared dough and cooked using an oven (9620I; Arcelik, Istanbul, Turkey) at 180 °C for 15 min.

### Proximate analysis

The moisture content was determined by oven drying (MST-120; Mikrotest, Ankara, Turkey) at 105 °C for 8 h according to AOAC Method 950.46 (*16*). The ash content was determined by igniting a weighted sample in a muffle furnace (ThermoStable OF-50; Daihan Scientific, Gangwon-do, Republic of Korea) at 550 °C to a constant mass according to AOAC Method 920.153 (*17*). A Soxhlet system (WHM 12293; Daihan Scientific, Gangwon-do, Republic of Korea) was used to extract the fat content with petroleum ether according to AOAC Method 991.36 (*18*). The protein amount was obtained by subtracting the sum of the other components (moisture, ash and oil) from 100. The energy value of meatballs was calculated by summing up the caloric values of proteins (16.74 kJ), fat (37.66 kJ) and carbohydrates (16.74 kJ).

### Instrumental determination of colour

The colour of raw and cooked meatball samples was determined by CIELAB colorimetric system using a hand-held colour analyzer (Ci64; X-rite, Grand Rapids, MI, USA). Luminosity, L*, a* and b* coordinates were considered for determination of the colour of samples. Also, the observer's angle was 2°, illuminator was D65/10 and its aperture size was 8 mm with the measurement area of 8 mm. The colorimeter was calibrated using white and black plaque prior to the measurements. After calibration, the average values were obtained of two parallel measurements at three different spots on the samples.

### Technological analysis

All samples in the same treatment group were cooked in the same oven (9620I; Arcelik) simultaneously to avoid exposure to temperature fluctuations and to minimize variation between the replications of each treatment. The same oven and the same temperature adjustments were applied in a controlled manner for each cooking. Technological analyses were carried out according to Heck *et al.* (*13*). Two replicates of three meatballs prepared from each sample were analyzed. The diameter and mass of raw samples were measured. The same steps were repeated after cooking and cooling the samples to 25 °C.

Cooking loss, diameter reduction and meat and fat retention of the meatballs were determined using the following equations:

*w*(cooking loss)=[(*m*(raw meatball)-*m*(cooked meatball))/*m*(raw meatball]•100 /1/

*d*_reduction_=[(*d*_raw meatball_-*d*_cooked meatball_)/*d*_raw meatball_]∙100 /2/

*w*(moisture retention)=[100-*w*(cooking loss)∙*w*(moisture of cooked meatball)]/·100 /3/

*w*(fat retention)=[(*m*(cooked meatball)∙*w*(cooked meatball lipid)/(*m*(raw meatball)∙*w*(raw meatball lipid)]∙100 /4/

### Fatty acid profile

Lipids were extracted according to the method described by Bligh and Dyer (*19*). The fatty acid composition was then determined by AOAC Method 996.01 (*20*). The fatty acid methyl esters (FAME) were analyzed by gas chromatography-flame ionisation detector (GC-2025; Shimadzu, Tokyo, Japan). In the analysis, nitrogen was used as a carrier gas at a flow rate of 30 mL/min, hydrogen as a combustible gas at 28 mL/min and dry air at 220 mL/min. The samples were injected into the device at 1 μL. The injector, column and detector temperatures were 200, 180 and 200 °C, respectively. Silica capillary column (RTX-2330) was used with 100 m×0.25 mm i.d. and film thickness 0.20 μm. Support material of the column was Chromosorb W(AW-DMCS) (60–80 mesh). Flame ionization detector (FID) was used for quantitative determination of components. The total analysis time was 52 min for each sample. The fatty acid profile of the samples was determined in triplicate.

### Lipid oxidation (TBARS)

The method of Pikul *et al*. (*21*) was used to determine lipid oxidation. A mass of 10 g of meatballs was mixed with 35 mL of 4 % HClO_4_. The meat and the perchloric acid were homogenized at 13 800 rpm. After filtering, 5 mL of distilled water were used to wash the slurry. A volume of 50 mL of the filtrate was added to HClO_4_. Equal volumes (5 mL) of filtrate and TBA (0.02 M) were mixed and left in a water bath at 80 °C for 60 min. The mixture tubes were cooled to room temperature and the absorbance was read by UV/Vis spectrophotometer (Optizen POP Series UV/Vis; Mecasys, Seoul, Republic of Korea) at 532 nm. The results were expressed in mg malondialdehyde (MDA) per kg meatball sample using the calibration curve prepared with tetramethoxypropane (TMP).

### Texture profile analysis

A texture analyzer (TA.HDplus; Stable Micro Systems Ltd, Godalming, UK) was used for the texture profile analysis, which was conducted with a 25 kg load cell (*22*). The samples were analyzed at room temperature and had a height of about 2 cm. The probe was placed 10 mm from the meatball and the test speed was 5 mm/s. The P/36R probe squeezed the meatball twice and its size was reduced by 50 %. The software of the instrument assessed the samples for resilience, gumminess, chewiness, adhesiveness, hardness and springiness.

### Statistical analysis

The study was carried out in two replications and two parallel analyses were performed in each replication. Data were analyzed with a general linear model at a significance level of 5 % using SPSS v. 27 (*23*). The Shapiro-Wilk test was used to determine the normality of the data distribution (*24*). The data are given as a mean value and a standard deviation. The Tukey’s test was used to determine the difference in the mean values if the distribution was normal and Dunnett's T3 test was used to determine the difference in the mean values if the distribution was not normal.

## RESULTS AND DISCUSSION

### The results of proximate analyses and technological parameters

The mass fractions of moisture, protein, fat and ash of minced meat used in the study were (76.3±0.2), (18.7±1.8), (377±1.8) and (1.3±0.2) %, respectively. The analysis results of the raw and cooked meatballs are given in Table 1. The gel affected the overall composition of raw and cooked meatballs, comparable to raw and cooked patties (*25*). Moisture mass fraction of raw meatball samples C, F25, F50, F75 and F100 was (53.7±2.0), (60.3±1.9), (64.0±0.3), (67.90±0.02) and (73.85±0.07) %, respectively (p<0.001). As the fat replacement increased, the moisture mass fractions also increased. As it was expected, and stated by Alejandre *et al.* (*25*), the moisture content of the reformulated meatballs increased significantly compared to the control group of the uncooked samples due to the higher water content in the gel (p<0.001). In addition, the same results were observed in similar studies using the gel system to reshape meatballs (*11**,**12*). As a result of the cooking process, there was no difference in the moisture mass fraction of the meatball samples.

**Table 1:** Results of the analysis of raw and cooked meatballs

Meatball sample	Group	*w*/%							*E*/kJ
Moisture	Fat		Protein		Ash	
Raw	C	(53.7±2.0)^d^		(31.2±227)^a^		(12.2±0.2)^a^		3.0±0.1	(2756±242)^a^
	F25	(60.3±1.9)^c^		(22.98±0.04)^b^		(13.75±2.05)^abc^		3.01±0.09	(2191±97)^b^
	F50	(64.0±0.3)^bc^		(1476±0.4)^c^		(18.4±0.1)^b^		2.88±0.01	(1721±50)^bc^
	F75	(67.90±0.02)^b^		(12.0±0.1)^cd^		(17.1±0.1)^c^		2.94±0.02	(1475±17)^cd^
	F100	(73.85±0.07)^a^		(10.5±0.4)^d^		(12.7±0.4)^abc^		2.92±0.02	(1217±62)^e^
	Sig	***		***		**		ns	**
Cooked	C	59.65±0.07		(8.7±1.2)^b^		(27.4±1.1)^ab^		4.27±0.01	1570±182
	F25	59.1±0.4		(10.14±0.04)^b^		(26.7±0.5)^ab^		3.99±0.02	1658±26
	F50	57.6±1.1		(10.0±0.8)^b^		(29.0±1.8)^a^		3.49±0.05	1659±254
	F75	57.4±0.1		(10.48±0.02)^b^		(28.7±0.1)^ab^		3.40±0.3	1750±8
	F100	58.4±1.2		(13.2±0.4)^a^		(25.0±0.2)^b^		3.4±0.6	1828±44
	Sig	ns		***		*		ns	ns

C=control, F25, F50, F75 and F100=samples with 25, 50, 75 and 100 % fat substitution, respectively. Different lowercase letters in superscript represent significant differences between the mean±S.D. Sig=significance, ns=not significant, *p<0.05, **p<0.01, ***p<0.001

When compared to the control group, the fat mass fraction of raw meatball samples with the highest fat substitute (F100) was reduced by approximately a third. Considering the oil content, the use of oil emulsion hydrogels has been reported to significantly reduce the fat mass fraction in dry-fermented foal sausages (*26*). Comparing the fat mass fractions of the cooked samples, it was determined that F100 sample had the highest oil content. Except for F100, the difference between the other samples was not significant (p>0.05). The fat mass fraction in the control group was found to be proportionally lower due to the cooking process, the melting oil and leaking matrix. Similarly, Salcedo-Sandoval *et al.* (*12*) replaced pork fat in frankfurters with oil, O/W emulsion or filled hydrogel and found the highest fat content in frankfurters with filled hydrogels.

Although Cittadini *et al.* (*26*) reported that there were no differences in the protein content between the samples, in our study, we determined differences between the protein mass fractions of raw and cooked samples in various ways. The differences in ash mass fraction between the raw and cooked control and the reformulated meatballs were relatively small and were not significant (p>0.05).

The fat retention was the highest in sample F100, which together with F75 had the highest cooking loss (Table 2). Consequently, the cooking loss of sample F100 can be attributed to considerable initial water content. This sample also had the highest moisture mass fraction among the uncooked samples. Similarly, although the samples in the control group had a lower cooking loss than the other samples, they had the highest water retention and the lowest oil retention. The cooking loss is probably related to the fat loss. There was no statistically significant difference between the samples in terms of diameter reduction (p>0.05). Hanula *et al.* (*27*) reported that the highest cooking loss was in the control group. The reason for our opposite result is that the hydrogel used is lyophilized and has a low moisture content, so the loss is lower and at the same time the moisture is retained in the samples with substituted fat content.

**Table 2:** Technological parameters of the cooked meatball samples

Group	*d*_reduction_/%	Cooking loss	*w*/% Moisture retention	Fat retention
C	21.2±7.0	(38.7±2.4)^d^	(36.5±1.4)^a^	(18.7±0.7)^e^
F25	29.4±5.4	(45.8±3.0)^c^	(32.0±1.8)^b^	(23.8±1.3)^d^
F50	26.3±4.3	(48.3±1.7)^bc^	(29.8±1.0)^bc^	(37.8±1.2)^c^
F75	27.0±4.5	(51.3±1.0)^a^	(28.0±0.6)^d^	(42.5±0.9)^b^
F100	26.7±4.6	(50.4±0.3)^ab^	(28.9±0.2)^c^	(59.3±0.4)^a^
Sig	ns	***	***	***

C=control, F25, F50, F75 and F100=sample with 25, 50, 75 and 100 % fat substitution, respectively. Different lowercase letters in superscript show significant differences between the mean±S.D. Sig=significance, ns=not significant, ***p<0.001

The energy values of the raw meatball samples (Table 1) decreased significantly when the substitution ratio increased. The energy values of sample F100 were almost 56 % lower than those of the control group. When analyzing the energy content of the cooked samples, no statistically significant difference was found between the mean values. It was believed to be because the oil retention values of sample F100 were also significantly higher than those of the control group, although the moisture retention rates of the control group samples were significantly higher than those of the F100 group. Furthermore, the results show that the energy values of the cooked meatball samples have lower energy from fat and higher energy from protein.

### Instrumental colour results

**Fig. 1:**
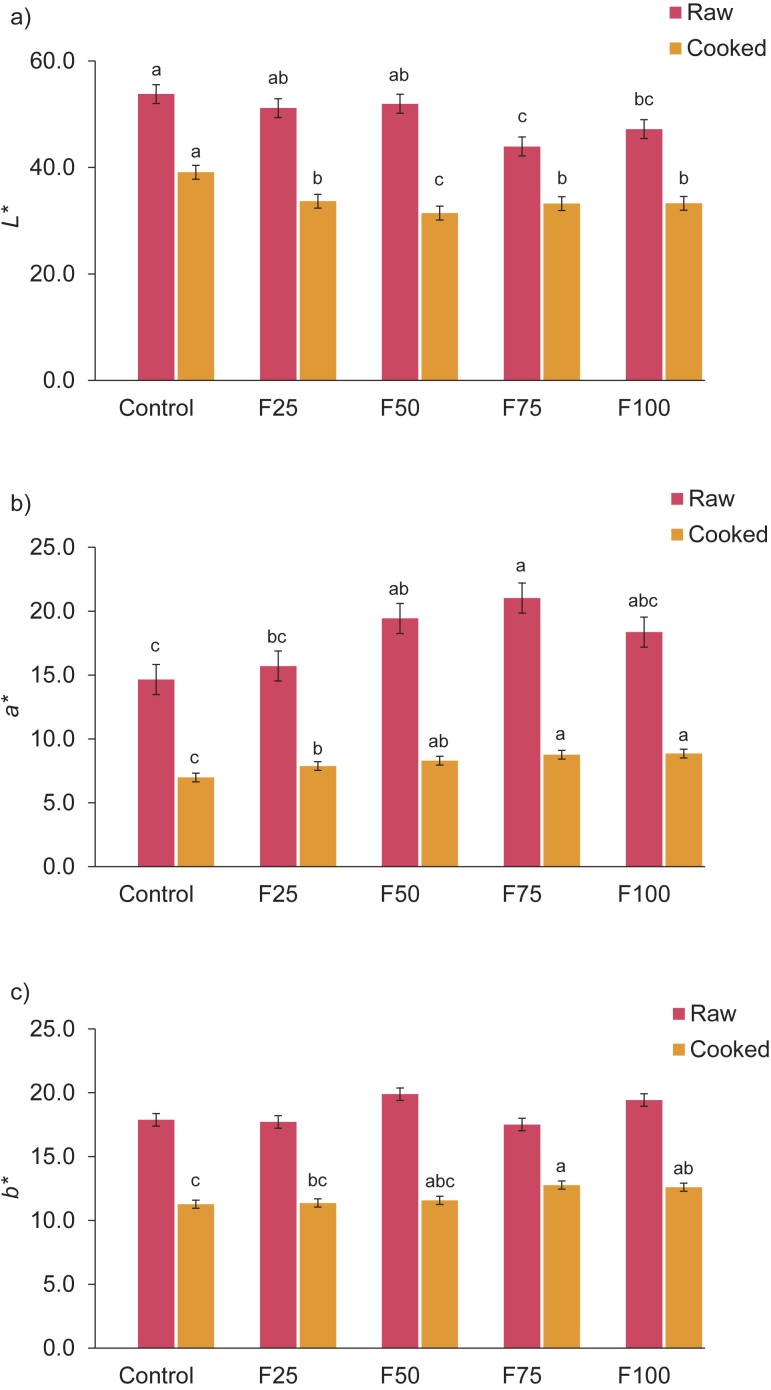
Results of colour measurements of the raw and cooked meatball samples: a) L* value, b) a* value, and c) b* value. F25, F50, F75 and F100=sample with 25, 50, 75 and 100 % fat substitution, respectively. Different lowercase letters in superscript show significant differences between the mean±S.D.

Kouzounis *et al.* (*28*) proposed that various fat sources had a partial impact on the instrumental colour values of sausages. The L*, a* and b* results of raw and cooked meatball samples are shown in Fig. 1. There was no statistically significant difference between the Δ*E* results of raw and cooked meatball samples (data not shown). The L* value decreased significantly (p<0.01) when the amount of hydrogel utilized in the raw meatballs increased, as seen in Fig. 1. Similar to the findings of Cittadini *et al.* (*26*), the samples with different amounts of fat substituted with hydrogels were found to be darker than the control group. Kouzounis *et al.* (*28*) reported that there were no significant differences in lightness and yellowness of frankfurters compared to control and fat-replaced samples, while the control group frankfurters had an intense red colour. According to Barbut *et al.* (*29*), treatments with organogels resulted in lower lightness values than of the control, and a* and b* values were not affected by the addition of organogel. The raw meatball samples with hydrogel had no differences in redness values, which were slightly higher than of the control group, while the difference between the control and test groups was not significant except for F50 and F75. It can be said that the differences in colour values are due to many factors such as the differences in the raw materials used, differences in the types of products produced, in the features of the oil substitutes used, cooking methods, *etc.* According to Salcedo-Sandoval *et al.* (*12*), the colour variations in the sausages depended on the system used to stabilize the oil-in-water emulsions made as fat substitutes, as well as on the colours of the fish oil and pork back fat used. Likewise, Delgado-Pando *et al.* (*30*) reported that the type of sausage, product formulation, characteristics of the oils and the tested fat delivery system mainly affected the colour of the products with fat substituted with different lipid sources.

### Profile of fatty acids

The profile of the fatty acid content of the raw and cooked meatballs is shown in Table 3. When comparing the fatty acid composition of the raw meatball samples with the fat-substituted samples, both increasing and decreasing values were observed. The results show that myristic, palmitic and stearic acids from saturated fatty acids were abundant in both the control group and the fat-substituted raw meatballs. The substituted groups had significantly (p˂0.05) lower amounts of total saturated fatty acids than the control group. These results are similar to Heck *et al.* (*13*). The total amount of saturated fatty acids decreased when the substitution ratio (F25, F50, F75 and F100) increased. Similar to the results shown in the study by Cittadini *et al.* (*26*), MUFA were the dominant group in all formulations in our study, followed by SFA and finally PUFA. The control group of cooked meatballs and the fat-substituted samples were also rich in saturated fatty acids, namely myristic, palmitic and stearic acids. The cooked meatballs contained more saturated fatty acids than the control group and the other F25 samples ((2929±3) mg/100 g). The total amount of saturated fatty acids was found to decrease with increasing substitution ratio (F50, F75 and F100). In addition, a decrease in saturated fatty acids was observed in the meatball samples due to oil loss, leakage and the cooking process of the meatballs.

**Table 3:** Fatty acid composition of the raw and the cooked meatballs

Fatty acid	*w*/(mg/100 g)									
Raw					Cooked				
Control	F25	F50	F75	F100	Control	F25	F50	F75	F100
C8:0	(44.3±1.8)^a^	0.0±0.0	(29.61±0.06)^b^	(9.7±0.1)^d^	(22.2±0.4)^c^	(3.81±0.06)^d^	(1.93±0.05)^e^	(13.55±0.00)^b^	(11.3±0.2)^c^	(15.0±0.4)^a^
C11:0	(36.8±0.8)^a^	(30.6±0.6)^b^	(6.4±0.3)^c^	(7.3±0.1)^c^	(0.42±0.04)^d^	(4.24±0.04)^c^	(2.23±0.03)^d^	(5.6±0.2)^b^	(7.2±0.2)^a^	(2.6±0.1)^d^
C14:0	(1043±4)^a^	(989±1)^b^	(476±4)^c^	(325±1)^d^	(189.0±0.4)^e^	(208.45±0.5)^c^	(469.5±0.75)^a^	(299.2±1.0)^b^	(209.3±0.2)^c^	(205.3±0.3)^c^
C16:0	(666±1)^a^	(515±2)^b^	(339±1)^c^	(18.36±0.07)^e^	(201.7±1.2)^d^	(4.85±0.01)^e^	(307.6±0.8)^a^	(271.6±0.1)^b^	(179.5±0.9)^d^	(225.1±0.3)^c^
C17:0	(9276±0.5)^a^	(77.7±0.4)^b^	(29.8±1.4)^d^	(44.9±0.6)^c^	(29.4±0.3)^d^	(34.0±0.1)^c^	(9.1±0.2)^d^	(37.2±0.8)^b^	(37.4±0.3)^b^	(46.5±0.5)^a^
C18:0	(9188±3)^a^	(5856±4)^b^	(3794±5)^c^	(2979.8±1.9)^d^	(1494±4)^e^	(2518.4±0.4)^a^	(2131±2)^b^	(1818±2)^c^	(1794.5±0.1)^d^	(1468±2)^e^
C20:0	(73.6±0.1)^a^	(48.3±0.5)^b^	(28.2±0.3)^d^	nd	(29.8±0.2)^c^	(1.30±0.03)^e^	(7.7±0.2)^d^	(23.1±1.5)^c^	(28.5±1.1)^b^	(51.1±0.4)^a^
∑SFA	(11143±3)^a^	(7514±1)^b^	(4700±1)^c^	(3385±2)^d^	(1967±2)^e^	(2775±4)^b^	(2929±3)^a^	(2468±3)^c^	(2267.8±3.0)^d^	(2014±2)^e^
C14:1	(135.1±1.7)^a^	(86.6±0.4)^b^	(59.52±0.07)^c^	(23.8±0.9)^e^	(33.11±0.08)^d^	(16.1±0.4)^e^	(58.4±0.7)^a^	(45.7±0.1)^b^	(28.4±0.2)^d^	(35.3±0.6)^c^
C15:1	(7966±5)^a^	(6258±3)^b^	(3652±1)^c^	(2934±2)^d^	(2218±0.2)^e^	(2144±3)^e^	(2820±2)^a^	(2503.8±0.4)^c^	(2281±1)^d^	(2626±2)^b^
C16:1	(440±2)^a^	(288.2±0.8)^b^	(186.0±1.9)^c^	(146.5±0.8)^d^	(72.1±0.7)^e^	(114.8±1.0)^a^	(113.77±0.03)^a^	(99.1±0.5)^b^	(94.32±0.03)^c^	(72.8±0.2)^d^
C18:1n9t	(1019±2)^a^	(580±3)^b^	(330.9±0.5)^c^	(267.0±0.3)^d^	(105.4±0.6)^e^	(296.0±0.7)^a^	(179.8±1.2)^b^	(146.0±1.5)^d^	(155.4±1.1)^c^	(93.8±0.2)^e^
C18:1n9c	(9714±2)^a^	(7571±8)^b^	(5201±4)^d^	(4676±4)^e^	(5370±4)^c^	(2965±1)^e^	(3726±4)^d^	(4218±1)^c^	(4962±2)^b^	(7477±2)^a^
C20:1	(126.7±1.1)^a^	(67.1±1.4)^b^	(51.9±0.4)^c^	nd	(22.4±0.4)^d^	(2.94±0.04)^cd^	(3.45±0.05)^c^	(20.9±0.1)^a^	(18.9±0.5)^b^	(1.712±0.08)^d^
∑MUFA	(19403±2)^a^	(14851±3)^b^	(9482±1)^c^	(8048±2)^d^	(7822±2)^e^	(5537±1)^e^	(6901±2)^d^	(7033±2)^c^	(7540±3)^b^	(10307±4)^a^
C18:2n6t	(34.3±0.2)^a^	(9.19±0.01)^b^	(12.3±0.3)^b^	(35.9±3.2)^a^	(0.63±0.04)^c^	(20.1±0.2)^b^	(1.93±0.06)^d^	(6.28±0.02)^c^	(103.5±0.5)^a^	(5.14±0.05)^c^
C18:2n6c	(435±1)^b^	(420±2)^c^	(383±1)^d^	(415.4±0.5)^c^	(64668±0.8)^a^	(190.4±2.5)^e^	(304±1)^d^	(412±1)^c^	(525±2)^b^	(802±1)^a^
C18:3n6c	(166.6±1.7)^b^	(185.7±1.6)^a^	(74.2±1.2)^c^	(71.0±056)^c^	(73.4±0.5)^c^	(51.9±0.2)^a^	(2.33±0.09)^e^	(30.6±0.3)^d^	(32.3±0.7)^c^	(35.0±0.2)^b^
C18:3n6	(17.8±0.2)^b^	nd	(8.4±0.1)^c^	(44.3±0.5)^a^	(1.16±0.02)^d^	(36.20±0.05)^a^	(1.83±0.04)^d^	(9.86±0.04)^c^	(11.1±1.1)^b^	(7.6±0.1)^c^
∑PUFA	(654±2)^b^	(615±1)^c^	(478±1)^e^	(567±2)^d^	(721±2)^a^	(299±1)^e^	(310±1)^d^	(459±2)^c^	(672±1)^b^	(850±1)^a^
∑UFA	(20057±5)^a^	(15466±3)^b^	(9959±2)^c^	(8614±3)^d^	(8543±3)^e^	(5836±2)^e^	(7211±2)^d^	(7492±3)^c^	(8212± 2)^b^	(11156±4)^a^

C=control, F25, F50, F75 and F100=sample with 25, 50, 75 and 100 % fat substitution, respectively. nd=not detected. SFA=saturated fatty acid, MUFA=monounsaturated fatty acid, PUFA=polyunsaturated fatty acid, UFA=unsaturated fatty acid. Different lowercase letters in superscript in the same row represent statistically significant differences between the mean±S.D. of the raw and cooked samples (p<0.01)

When the composition of unsaturated fatty acids of the raw meatball samples was examined, it was found that both the control group and the processed samples had high amounts of the monounsaturated fatty acids such as oleic acid, *cis*-10-pentadecanoic acid and elaidic acid. High mass fractions of linoleic acid were also observed. Compared to the control group and the other samples, sample F25 had a high mass fraction of total monounsaturated fatty acids ((14851±3) mg/100 g), while sample F100 had a high mass fraction of total polyunsaturated fatty acids ((722±2) mg/100 g). As in our study, Heck *et al.* (*13*) found that in raw and cooked burgers the most common MUFA content was oleic acid (18:1n-9c) and the most common PUFA content was linoleic acid (C18:2n6c). The total monounsaturated fatty acid mass fraction decreased as the fat substitution from F25 to F100 increased. The raw meatballs of the control group had a total MUFA mass fraction of (19403±2) mg/100 g and a total PUFA mass fraction of (654±2) mg/100 g.

### Lipid oxidation (TBARS) results

**Fig. 2:**
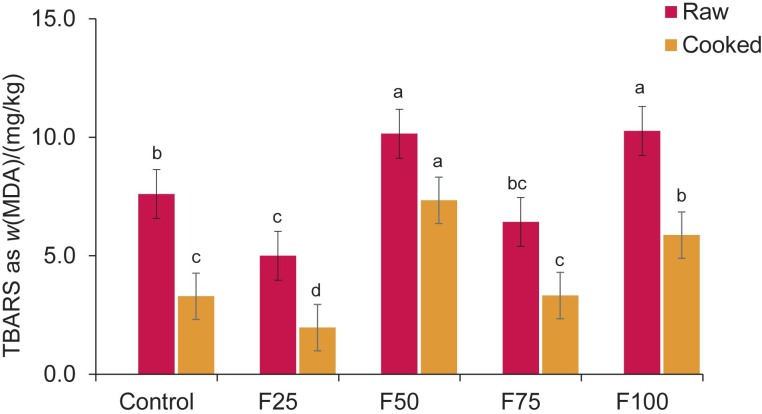
Results of lipid oxidation of the raw and cooked meatball samples. F25, F50, F75 and F100=sample with 25, 50, 75 and 100 % fat substitution, respectively. TBARS=thiobarbituric acid reactive substances, MDA=malondialdehyde. Different lowercase letters in superscript show significant differences between the mean±S.D.

Fig. 2 shows the differences in the results of lipid oxidation of the raw and cooked meatballs. Among the raw meatball samples, the highest oxidation occurred in samples F50 and F100. However, it was found that the difference between F25 and F75 and the difference between control and F75 were not statistically significant (p˃0.05). When the cooked samples were compared, sample F50 had the highest TBA value, followed by F100 and F75. The lack of a linear relationship can be attributed to the fact that hydrogel in the meatball sample is not homogeneously distributed. However, the fatty acid compositions also show that as the hydrogel substitution rate increases, the amount of oxidation degradation products is likely to be higher due to the increase in the amount of monounsaturated and polyunsaturated fatty acids (*22*).

### Texture profile analysis results

The results of hardness, springiness, cohesiveness, gumminess, chewiness and resilience of the meatballs are given in Table 4. In this study, the reformulation of the lipid profile of meatballs with gelatinized olive oil did not affect the springiness and cohesiveness of the samples (p˃0.05). The hardness of samples decreased by at least 25 % when the fat was replaced. Heck *et al.* (*13*) suggested that the hardness also increased when the protein/lipid ratio in the burgers increased. In this study, it was found that the hardness was lower in the samples with the highest substitution ratio although the protein/lipid ratio of the cooked samples increased, contrary to what they reported. The hardness values of the control, F25, and F50 did not differ significantly from each other, but they were different from F75 and F100 (p˂0.05). This could be due to structural differences between the oil substitutes used in the study, the lower oil content of the hydrogel sample and the fact that oil retention is highest in the samples with the highest oil replacement rate and the lowest water retention. Moghtadaei *et al.* (*31*) found that oleogels have lower hardness than animal fats, which significantly reduces the hardness, stickiness and chewiness of hamburgers compared to the control sample. Özer and Çelegen (*32*) also reported that replacing animal fat with an oleogel-based emulsion in beef burgers resulted in lower hardness, chewiness and springiness of the samples. Barbut *et al.* (*29*) reported that fat replacement of pork breakfast sausages with canola oil organogels reduced hardness values. These conditions affect the gumminess, chewiness and resilience values of the meatball samples. They concluded that these differences were because the hydrogels used as a fat substitute are structurally softer than adipose tissue and the distribution within the samples was not uniform.

**Table 4:** Texture profile analysis results of the cooked meatballs

Group	Hardness/N	Springiness/mm	Cohesiveness	Gumminess/g	Chewiness/(g/mm)	Resilience
C	(19.4±3.00)^a^	1.1±0.2	0.84±0.04	(1549±286)^ab^	(1474±222)^abc^	(0.62±0.04)^b^
F25	(19.3±2.5)^a^	0.99±0.01	0.83±0.01	(1580±199)^a^	(1543±190)^ab^	(0.62±0.04)^b^
F50	(19.1±1.6)^a^	1.00±0.01	0.84±0.01	(1536±202)^ab^	(1642±1489)^a^	(0.65±0.03)^ab^
F75	(15.3±1.7)^b^	1.00±0.01	0.85±0.01	(1247±167)^bc^	(1247±168)^bc^	(0.65±0.03)^ab^
F100	(14.3±2.0)^b^	0.99±0.09	0.86±0.01	(1150±242)^c^	(1144±274)^c^	(0.68±0.03)^a^
Sig	***	ns	ns	**	**	**

C=control, F25, F50, F75 and F100=sample with 25, 50, 75 and 100 % fat substitution, respectively. Different lowercase letters in superscript show significant differences between the mean±S.D. Sig=significance, ns=not significant, **p<0.01, ***p<0.001

## CONCLUSIONS

It is important to choose the correct fat replacement ratio, as we did in our study in accordance with the data from the literature. The use of the optimal replacement ratio enables the production of products that meet consumer requirements without affecting the technological and quality properties of the meatballs. The limitation of this study is the lack of consumer evaluation and storage process. In future studies, ensuring the optimal replacement ratio and meeting consumer preferences should be supported by sensory analyses. Furthermore, monitoring the changes of the hydrogel throughout the storage period is crucial for the development of effective measures to optimise the hydrogel. In the development of products with a healthier fatty acid profile, the use of hydrogels produced from gelatinized olive oil emulsion can be beneficial as a fat substitute in meatballs. This research is important to get an idea for new studies that are being planned to improve the technological properties of hydrogels with healthier lipid formulations and to expand their use in this area. The samples with 25 % hydrogel addition showed the most similar values, although they had higher cooking loss, oil retention and decreased water retention capacity than the control group. In addition to these properties, the texture study showed no differences between the values of hardness, gumminess, chewiness and resilience of the control group. At this rate of substitution, the energy value of cooked meatballs was reduced by about 20 %. The amounts of mono- and polyunsaturated fatty acids significantly increased compared to the control group. In addition, the group of meatballs in which 25 % fat was substituted had the lowest thiobarbituric acid amounts. It is believed that a 25 % replacement with hydrogel would be suitable for the composition of the meatballs.
